# Mutual effect of homocysteine and uric acid on arterial stiffness and cardiovascular risk in the context of predictive, preventive, and personalized medicine

**DOI:** 10.1007/s13167-022-00298-x

**Published:** 2022-09-26

**Authors:** Zhiyuan Wu, Haiping Zhang, Zhiwei Li, Haibin Li, Xinlei Miao, Huiying Pan, Jinqi Wang, Xiangtong Liu, Xiaoping Kang, Xia Li, Lixin Tao, Xiuhua Guo

**Affiliations:** 1 Beijing Municipal Key Laboratory of Clinical Epidemiology, Department of Epidemiology and Health Statistics, School of Public Health, Capital Medical University, No. 10 Xitoutiao, Youanmen Street, Beijing, 100069 China; 2 Centre for Precision Health, School of Medical and Health Sciences, Edith Cowan University, Perth, Australia; 3 Heart Center & Beijing Key Laboratory of Hypertension, Beijing Chaoyang Hospital, Capital Medical University, Beijing, China; 4 Beijing Xiaotangshan Hospital, Beijing, China; 5 Department of Mathematics and Statistics, La Trobe University, Melbourne, Australia

**Keywords:** Predictive preventive and personalized medicine (PPPM / 3PM), Arterial stiffness, Brachial-ankle pulse wave velocity, Homocysteine, Uric acid, Cardiovascular risk, Mutual effect

## Abstract

**Background:**

Arterial stiffness is a major risk factor and effective predictor of cardiovascular diseases and a common pathway of pathological vascular impairments. Homocysteine (Hcy) and uric acid (UA) own the shared metabolic pathways to affect vascular function. Serum uric acid (UA) has a great impact on arterial stiffness and cardiovascular risk, while the mutual effect with Hcy remains unknown yet. This study aimed to evaluate the mutual effect of serum Hcy and UA on arterial stiffness and 10-year cardiovascular risk in the general population. From the perspective of predictive, preventive, and personalized medicine (PPPM/3PM), we assumed that combined assessment of Hcy and UA provides a better tool for targeted prevention and personalized intervention of cardiovascular diseases via suppressing arterial stiffness.

**Methods:**

This study consisted of 17,697 participants from Beijing Health Management Cohort, who underwent health examination between January 2012 and December 2019. Brachial-ankle pulse wave velocity (baPWV) was used as an index of arterial stiffness.

**Results:**

Individuals with both high Hcy and UA had the highest baPWV, compared with those with low Hcy and low UA (*β*: 30.76, 95% CI: 18.36–43.16 in males; *β*: 53.53, 95% CI: 38.46–68.60 in females). In addition, these individuals owned the highest 10-year cardiovascular risk (OR: 1.49, 95% CI: 1.26–1.76 in males; OR: 7.61, 95% CI: 4.63–12.68 in females). Of note, males with high homocysteine and low uric acid were significantly associated with increased cardiovascular risk (OR: 1.30, 95% CI: 1.15–1.47), but not the high uric acid and low homocysteine group (OR: 1.02, 95% CI: 0.90–1.16).

**Conclusions:**

This study found the significantly mutual effect of Hcy and UA on arterial stiffness and cardiovascular risk using a large population and suggested the clinical importance of combined evaluation and control of Hcy and UA for promoting cardiovascular health. The adverse effect of homocysteine on arteriosclerosis should be addressed beyond uric acid, especially for males. Monitoring of the level of both Hcy and UA provides a window opportunity for PPPM/3PM in the progression of arterial stiffness and prevention of CVD. Hcy provides a novel predictor beyond UA of cardiovascular health to identify individuals at high risk of arterial stiffness for the primary prevention and early treatment of CVD. In the progressive stage of arterial stiffness, active control of Hcy and UA levels from the aspects of dietary behavior and medication treatment is conducive to alleviating the level of arterial stiffness and reducing the risk of CVD. Further studies are needed to evaluate the clinical effect of Hcy and UA targeted intervention on arterial stiffness and cardiovascular health.

**Supplementary Information:**

The online version contains supplementary material available at 10.1007/s13167-022-00298-x.

## Introduction

### Arterial stiffness is a major predictor of cardiovascular disease

Arterial stiffness, which reflects arterial elasticity and function, increases with age and is affected by health conditions such as diabetes and hypertension [1–3]. Arterial stiffness is becoming more prevalent as the population aging and the increased prevalence of chronic diseases. Previous studies have discussed the role of arterial stiffness in the pathophysiology of cardiovascular diseases [4]. In addition to causing vascular damages such as vascular structural changes and endothelial dysfunction, arterial stiffness is closely associated with increased cardiac load and decreased coronary perfusion pressure, microvascular ischemia, arterial wave reflections, renal dysfunction, and other cardiovascular risks [5, 6]. Population-based studies have shown that arterial stiffness is an independent risk factor and early predictor of cardiovascular diseases especially in the high-risk individuals with hypertension, diabetes, or metabolic syndrome [3, 6–8]. Thus, the early prevention and intervention of arterial stiffness, especially its unfavorable progression, are particularly crucial for suppressing the global health burden and promoting health quality. The predictive, preventive, and personalized medicine (PPPM/3PM) aims to develop predictive diagnostic tool, targeted prevention strategy, and personalized medical services to the population [9]. Novel biomarkers for early detection and preventing of arterial stiffness progression are needed from the standpoint of PPPM/3PM.

### The mutual effect of homocysteine and uric acid on arterial stiffness status is a bridge for cardiovascular disease prediction in the context of PPPM/3PM

As an integrative strategy in health care system, the PPPM/3PM aims to promote health care-related research and management based on multidisciplinary expertise via early prediction of disease predisposition, timely prevention of disease onset or progression, and personalized treatment of diseases outcomes [10]. Development of innovative screening method contributes to promote population health by health risk assessment, individualized, and cost-effective profiling. It has been highlighted that homocysteine (Hcy)/uric acid (UA) metabolism and cardiovascular diseases are several areas considered to be potentially strongly benefiting from the PPPM/3PM paradigm [11, 12]. Koklesova et al. reported that the metabolic pathway of Hcy is closely related to coronary artery disease and atherosclerosis, which is of great value for disease diagnosis and prediction, and helps to optimize the individualized comprehensive treatment of patients [11]. Numerous studies have confirmed that high homocysteine (Hcy) and high uric acid (UA) are important risk factors and predictors of cardiovascular events, including acute myocardial infarction, atherosclerosis, and stroke [13]. Both Hcy and UA can mediate vascular endothelial dysfunction by activating oxidative stress and inhibiting endothelial nitric oxide synthase (eNOS), and stimulate the proliferation of vascular smooth muscle cells, resulting in subclinical changes in arterial structure [14–17]. In addition, Hcy and UA are significantly correlated with arterial stiffness indicators, including carotid-radial pulse wave velocity (crPWV), brachial-ankle pulse wave velocity (baPWV), or carotid-femoral artery pulse wave velocity (cfPWV) [16, 18, 19]. Recently, Wang et al. reported the combined effect of Hcy and UA on subclinical atrial fibrillation (SCAF) [20]. Marković-Boras et al. found that the combined measurements of serum Hcy and UA can better assess the risk of acute myocardial infarction in patients with diabetes [21], indicating that Hcy and UA have a potential mutual effect on atherosclerosis. However, the combined effect of Hcy and UA on arterial stiffness and overall cardiovascular risk in the general population remains unknown yet. This study assumed that abnormal metabolism of Hcy and UA could lead to arterial stiffness by inducing vascular endothelial dysfunction and then increase the risk of cardiovascular disease. Exploration of mutual effect of Hcy and UA as biomarkers of arterial stiffness status could inform the high-risk population to adapt personalized behavior to moderate the cardiovascular health status from the aspects of PPPM/3 M.

### Aim of this study

From the perspective of PPPM/3 M, effective identification of risk factors for arterial stiffness is crucial for the lowering the health burden and adverse consequences. This study aims to investigate the mutual effect of serum Hcy and UA on arterial stiffness and 10-year cardiovascular risk using a cross-sectional design, and evaluate whether effects of homocysteine and uric acid on arterial stiffness and cardiovascular risk are distinct between males and females.

## Material and methods

### Study population and design

This cross-sectional study enrolled participants from Beijing Health Management Cohort (BHMC) from January 2012 to December 2019. Details of the cohort design have been described in previous study [22]. All participants undertook the questionnaire survey, physical examination, and routine laboratory tests. Finally, 17,697 individuals aged 20 years or above, and with required data were included in this study. This study was in accordance with the principles of the Declaration of Helsinki and was approved by the Ethics Committee of Capital Medical University (grant number: 2020SY031). All participants provided their written informed consents before participating in the current study.

### Data measurement and definitions

The demographic characteristics and lifestyle behaviors factors were acquired by standard questionnaire interview. Educational level was categorized as illiteracy or primary school, middle school, high school or above. Taking moderate or intense physical activity “ ≥ 20 min each time and ≥ 4 times per week” was regarded as regular exercise. Smoking status was divided into never or former, and current smoker. Individual with an average alcohol consumption of more than 30 g per day was considered as current drinker.

Physical examination information and laboratory tests data were acquired through the electronic information system. Body mass index (BMI) was calculated as weight (kg)/[height (m)*height (m)]. Systolic and diastolic blood pressures were calculated as the mean of two measurements using the mercury sphygmomanometer on the right arm after resting for at least 10 min. Laboratory tests were carried out using fasting venous blood samples including assays for triglyceride, total cholesterol, high density lipoprotein (HDL) cholesterol, low density lipoprotein (LDL) cholesterol, fasting plasma glucose, glycated hemoglobin (HbA1c), serum Hcy, and serum UA. According to a previous study based on the Chinese population, high level Hcy was defined as > 14 µmol/L in males and > 10 µmol/L in females, and high level UA was defined as > 420 µmol/L in males and > 320 µmol/L in females [20]. The definition of obesity was BMI ≥ 28 kg/m^2^ for the Chinese population [23]. Hypertension was defined as systolic pressure ≥ 140 mmHg or diastolic pressure ≥ 90 mmHg or use of any anti-hypertensive medication or self-reported diagnosis history of hypertension according to the JNC-7 report [24]. Diabetes was defined as fasting glucose ≥ 7.0 mmol/L, HbA1c ≥ 6.5%, or the use of any anti-diabetic medication or self-reported diagnosis history of diabetes based on the American Diabetes Association 2020 report [25]. According to the Guidelines on Prevention and Treatment of Dyslipidaemia for Chinese Adults, dyslipidemia was defined as triglycerides ≥ 2.3 mmol/L, total cholesterol ≥ 6.2 mmol/L, LDL cholesterol ≥ 4.1 mmol/L, HDL cholesterol < 1.0 mmol/L, or any lipid-lowering medication or self-reported diagnosis history of dyslipidemia [26]. Cardiovascular diseases were self-reported in this study, including coronary heart disease, myocardial infarction, and stroke. Information about use of anti-gout medication and aspirin were acquired.

### BaPWV assessment

The baPWV was automatically measured using Omron Colin BP-203RPE III device (Omron Health Care, Kyoto, Japan) as described previously [22]. After more than 5 min of rest in the supine position, 4 cuffs were wrapped around the bilateral brachia and ankles and then connected to a plethysmographic sensor and oscillometric pressure sensor. The time interval between the wave front of the brachial waveform and the ankle waveform was expressed as the time interval between the brachium and ankle (∆Tba). The final baPWV was calculated as baPWV = (La − Lb)/∆Tba. La was the path length from the suprasternal notch to the ankle, and Lb was the path length from the suprasternal notch to the brachium. The maximum value of baPWV measurements on left and right sides was obtained.

### Cardiovascular risk calculation

We used the Framingham Heart Study score to evaluate the overall 10-year cardiovascular risk [27]. The score includes age, sex, total cholesterol, HDL cholesterol, systolic blood pressure, blood pressure lowering medication use, diabetes status, and smoking status in the primary model for individuals from 30 to 74 years and without cardiovascular disease history. Individuals with 10-year cardiovascular risk ≥ 20% were grouped as high risk.

For males, the risk is calculated as 1 − 0.88936^exp(Σ*βX*−23.9802)^, where *β* is the regression coefficient and *X* is the level for each risk factor as following equation:

$$\Sigma\beta X\:=\:3.06117\ast\log(age)\:+\:1.12370\ast\log(total\;cholesterol\;\lbrack mg/dL\rbrack)\:-\:0.93263\ast\log(HDL\;cholesterol\;\lbrack mg/dL\rbrack)\:+\:0.65451\ast smoker\;\lbrack yes\;or\;no\rbrack\:+\:0.57367\ast diabetes\;\lbrack yes\;or\;no\rbrack\:+\:1.93303\ast\log(systolic\;pressure\;not\;treated)\;or\;1.99881\ast\log(systolic\;pressure\;treated).$$

For females, the risk is calculated as 1 − 0.95012^exp(Σ*βX*−26.1931)^ as following equation:

$$\Sigma\beta X\:=\:2.32888\ast\log(age)\:+\:1.20904\ast\log(total\;cholesterol\;\lbrack mg/dL\rbrack)\:-\:0.70833\ast\log(HDL\;cholesterol\;\lbrack mg/dL\rbrack)\:+\:0.52873\ast smoker\;\lbrack yes\;or\;no\rbrack\:+\:0.69154\ast diabetes\;\lbrack yes\;or\;no\rbrack\:+\:2.76157\ast\log(systolic\;pressure\;not\;treated)\;or\;2.82263\ast\log(systolic\;pressure\;treated).$$

### Statistical analysis

The statistical analyses were conducted using R software, version 4.1.0 (R Foundation). Considering the sex-specific differences in metabolism of Hcy and UA, all analyses were stratified by gender. The difference was considered statistically significant at two-side significance level of *P* < 0.05.

Baseline characteristics are presented as the mean (standard deviation, SD) or median [interquartile range, IQR] or number (proportions), as appropriate. Differences between males and females were compared using chi-square test for categorical variables, and Student’s *t* test or Mann–Whitney *U* test for continuous variables. Differences in baPWV level among groups divided by Hcy and UA were compared using Kruskal–Wallis test. The correlations between Hcy and UA were analyzed using Spearman’s coefficients.

Unadjusted and adjusted linear regression models were used to estimate the association of Hcy and UA concentrations with baPWV level. Age, education level, BMI, physical activity, current smoking, current drinking, hypertension or not, diabetes or not, dyslipidemia or not, systolic pressure, fasting glucose, triglyceride, and HDL cholesterol were considered in the adjusted analyses. We calculated the regression coefficient (*β*) and corresponding 95% confidence interval (CI) for one-SD increase of Hcy and UA, respectively. To investigate the mutual effect between Hcy and UA, the population were divided into four groups: low Hcy/low UA, high Hcy/low UA, low Hcy/high UA, and high Hcy/high UA by sex specific cutoffs. In addition, the associations of Hcy (UA) concentration with baPWV were presented in people with low and high UA (Hcy) level. Then, we analyzed the mutual effect of Hcy and UA in subgroups stratified by age, obesity, hypertension, diabetes, and smoking status. We performed multiple sensitivity analyses. First, participants with cardiovascular diseases were excluded from the analysis; second, we excluded the individuals using any following medications: anti-hypertensive, anti-diabetic, lipid-lowering, anti-gout, and aspirin, to explore the stability of our findings. In addition, we used the logistics regression models to evaluate the association of Hcy and UA groups with 10-year cardiovascular risk.

## Results

### Population characteristics

The final analysis enrolled a total of 17,697 participants, including 12,604 men and 5093 women. The mean age (± SD) was 58.95 (13.99) years in men and 54.81 (14.03) years in women. The medians [P_25_–P_75_] of serum Hcy were 11.8 [9.8 − 14.7] μmol/L for men and 9.0 [7.4–11.0] μmol/L for women; UA were 372 [323–423] μmol/L for men and 284 [243–330] μmol/L for women; baPWV were 1489 [1345–1713] cm/s for men and 1391 [1252–1595] cm/s for women. Table 1 shows the detailed characteristics according to sex, and characteristic distribution stratified by UA and Hcy is shown in Tables S1 and S2. The People with high Hcy and UA concentrations had the highest baPWV level, compared with low Hcy/low UA group, high Hcy/low UA group, and low Hcy/high UA group (Figure S1 A–B). For men, there was a significantly positive correlation between Hcy and UA levels (Spearman’s coefficient = 0.113; *P* < 0.001). Similar result was found for women (Spearman’s coefficient = 0.225; *P* < 0.001) as shown in Figure S1 C–D.

**Table 1:** Characteristic distribution of participants

	Males (*n* = 12,604)	Females (*n* = 5093)	*P* value
Age (years)	58.95 (13.99)	54.81 (14.03)	< 0.001
Education (*n*, %) ^a^			0.281
Primary school or below	1030 (8.2)	452 (8.9)	
Middle school	7545 (59.9)	3008 (59.1)	
High school or above	4029 (32.0)	1633 (32.1)	
Physical activity (*n*, %) ^b^	7229 (57.4)	3251 (63.8)	< 0.001
Current smoking (*n*, %)	3062 (24.3)	825 (16.2)	< 0.001
Current drinking (*n*, %)	6301 (50.0)	1858 (36.5)	< 0.001
BMI (kg/m^2^) ^c^	26.03 (3.07)	24.32 (3.50)	< 0.001
Obesity (*n*, %) ^d^	2777 (23.2)	678 (14.1)	< 0.001
SBP (mmHg)	128.24 (15.81)	119.86 (17.87)	< 0.001
DBP (mmHg)	73.01 (10.76)	67.99 (10.08)	< 0.001
Triglyceride (mmol/L)	1.36 [0.98,1.97]	1.14 [0.82,1.59]	< 0.001
Total cholesterol (mmol/L)	4.52 [3.90,5.17]	4.76 [4.20,5.39]	< 0.001
LDL-C (mmol/L)	2.97 [2.38,3.57]	3.06 [2.51,3.64]	< 0.001
HDL-C (mmol/L)	1.19 [1.03,1.41]	1.44 [1.20,1.72]	< 0.001
Fasting glucose (mmol/L)	5.37 [4.98,6.01]	5.06 [4.75,5.47]	< 0.001
HbA1c (%)	5.77 [5.48,6.19]	5.71 [5.46,6.00]	< 0.001
Health status (*n*, %)			
Hypertension	4192 (33.3)	1051 (20.6)	< 0.001
Diabetes	2166 (17.2)	421 (8.3)	< 0.001
Dyslipidemia	4761 (37.8)	1241 (24.4)	< 0.001
Cardiovascular diseases	627 (5.0)	144 (2.8)	< 0.001
Medication use (*n*, %)			
Antihypertensive	1734 (13.8)	441 (8.7)	< 0.001
Antidiabetic	719 (5.7)	139 (2.7)	< 0.001
Lipid lowering	1128 (8.9)	372 (7.3)	< 0.001
Anti-gout	60 (0.5)	3 (0.1)	< 0.001
Aspirin	439 (3.5)	95 (1.9)	< 0.001
Homocysteine (μmol/L)	11.8 [9.8,14.7]	9.0 [7.4,11.0]	< 0.001
Uric acid (μmol/L)	372.0 [323.0,423.0]	284.0 [243.0,330.0]	< 0.001
BaPWV (cm/s)	1489.0 [1345.0,1713.0]	1391.0 [1252.0,1595.0]	< 0.001

Data are presented as mean (SD), median [IQR], or number (%), as appropriate

Abbreviations: *SD* standard deviation, *IQR* interquartile range, *BMI* body mass index, *SBP* systolic blood pressure, *DBP* diastolic blood pressure, *HDL-C* high-density lipoprotein cholesterol, *LDL-C* low-density lipoprotein cholesterol, *HbA1c* glycated hemoglobin, *baPWV* brachial-ankle pulse wave velocity

^a^Education level was divided into primary school or below, middle school, and high school or above

^b^Physical activity refers to having moderate or intense exercise ≥ 80 min a week

^c^BMI was calculated as weight in kilograms divided by height in meters squared

^d^Obesity is confirmed by BMI ≥ 28 kg/m^2^

### Association of Hcy and UA with arterial stiffness

In the adjusted model, one-SD increase in Hcy was associated with a 7.38 (95% CI: 3.98–10.79) cm/s increase of baPWV in men and an 11.00 (95% CI: 6.33–15.68) cm/s increase of baPWV in women. Men with high Hcy (> 14 µmol/L) had a 14.19 (95% CI: 6.60–21.78) cm/s higher baPWV than those with low Hcy; women with high Hcy (> 10 µmol/L) had a 21.61 (95% CI: 11.71–31.51) cm/s higher baPWV. Similarly, 1-SD increase in UA was associated with a 7.65 (95% CI: 4.11–11.18) cm/s increase of baPWV in men and a 16.60 (95% CI: 11.56–21.63) cm/s increase of baPWV in women. Men with high UA (> 420 µmol/L) had a 14.74 (95% CI: 6.85–22.63) cm/s higher baPWV than those with low UA; women with high UA (> 320 µmol/L) had a 38.16 (95% CI: 27.51–48.81) cm/s higher baPWV. Of note, individuals with both high Hcy and high UA had a 30.76 (95% CI: 18.36–43.16) cm/s increase of baPWV in men compared with those with low Hcy and low UA, which was higher than those just with high Hcy (*β*: 10.80, 95% CI: 1.85–19.74) or high UA (*β*: 10.96, 95% CI: 1.41–20.51). Similarly, female individuals with both high Hcy and high UA had a 53.53 (95% CI: 38.46–68.60) cm/s increase of baPWV in men compared with those with low Hcy and low UA, which was higher than those just with high Hcy (*β*: 21.63, 95% CI: 9.73–33.53) or high UA (*β*: 39.77, 95% CI: 26.35–53.19) as shown in Table 2.

**Table 2:** Separate and mutual associations of serum Hcy and UA with baPWV in males and females

	Unadjusted model			Adjusted model ^a^		
	*β*	95% CI	*P* value	*β*	95% CI	*P* value
Males						
Hcy (Per-SD: 7.64 μmol/L)	25.372	20.349, 30.394	< 0.001	7.383	3.979, 10.788	< 0.001
Low Hcy (*n* = 8896)						
High Hcy (*n* = 3708)	94.471	83.530, 105.412	< 0.001	14.193	6.604, 21.782	< 0.001
UA (Per-SD: 78.65 μmol/L)	− 4.266	− 9.308, 0.776	0.097	7.648	4.113, 11.182	< 0.001
Low UA (*n* = 9294)						
High UA (*n* = 3310)	4.392	− 7.066, 15.849	0.453	14.740	6.847, 22.633	< 0.001
Low Hcy and low UA (*n* = 6731)						
High Hcy and low UA (*n* = 2563)	84.652	71.664, 97.641	< 0.001	10.795	1.846, 19.743	0.018
Low Hcy and High UA (*n* = 2165)	− 13.538	− 27.365, 0.288	0.055	10.958	1.405, 20.510	0.025
High Hcy and high UA (*n* = 1145)	105.779	87.890, 123.668	< 0.001	30.755	18.355, 43.156	< 0.001
Females						
Hcy (Per-SD: 3.98 μmol/L)	63.395	56.112, 70.677	< 0.001	11.003	6.328, 15.679	< 0.001
Low Hcy (*n* = 3300)						
High Hcy (*n* = 1793)	137.450	122.242, 152.657	< 0.001	21.612	11.711, 31.513	< 0.001
UA (Per-SD: 69.22 μmol/L)	84.683	77.569, 91.797	< 0.001	16.596	11.557, 21.634	< 0.001
Low UA (*n* = 3588)						
High UA (*n* = 1505)	165.384	149.624, 181.144	< 0.001	38.164	27.514, 48.814	< 0.001
Low Hcy and low UA (*n* = 2486)						
High Hcy and low UA (*n* = 1102)	110.475	92.321, 128.629	< 0.001	21.628	9.729, 33.527	< 0.001
Low Hcy and High UA (*n* = 814)	139.512	119.255, 159.769	< 0.001	39.766	26.347, 53.186	< 0.001
High Hcy and high UA (*n* = 691)	269.763	248.190, 291.335	< 0.001	53.527	38.457, 68.598	< 0.001

Abbreviations: *Hcy* homocysteine, *UA* uric acid, *baPWV* brachial-ankle pulse wave velocity

^a^Adjusted for age, education, BMI, physical activity, smoking, drinking, health status, systolic pressure, glucose, triglyceride, HDL-C

**Fig. 1:**
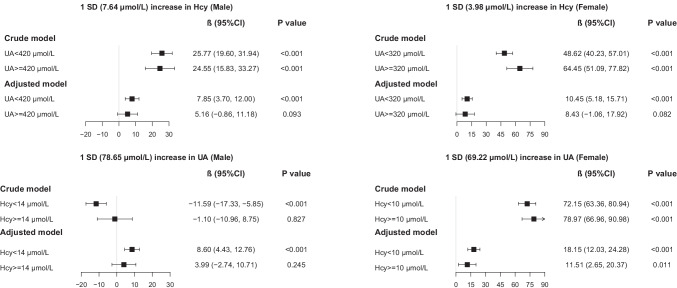
Adjusted associations of one-SD increase in Hcy/UA with baPWV stratified by UA/Hcy levels. Abbreviations: Hcy homocysteine, UA uric acid, baPWV brachial-ankle pulse wave velocity, SD standard deviation. The SD of UA and Hcy in males was 78.65 µmol/L and 7.64 µmol/L; SD of UA and Hcy in females was 69.22 µmol/L and 3.98 µmol/L. *β* refers to the regression coefficients in the multi-variable linear regression model after adjusted for age, education, BMI, physical activity, smoking, drinking, health status, systolic pressure, glucose, triglyceride, HDL-C

**Fig. 2:**
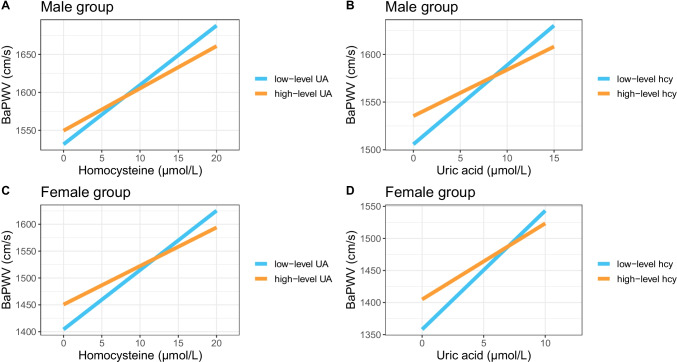
Adjusted linear regression lines of Hcy/UA and baPWV stratified by UA/Hcy levels. Abbreviations: Hcy homocysteine, UA uric acid, baPWV brachial-ankle pulse wave velocity. High level Hcy was defined as > 14 µmol/L in male and > 10 µmol/L in female; and high level UA was defined as > 420 µmol/L in male and > 320 µmol/L in female. The regression lines were plotted after adjusting for age, education, BMI, physical activity, smoking, drinking, health status, systolic pressure, glucose, triglyceride, HDL-C using R package ‘effects’. **A**: linear regression lines of Hcy and baPWV stratified by UA levels in male; **B**: linear regression lines of UA and baPWV stratified by Hcy levels in male; **C**: linear regression lines of Hcy and baPWV stratified by UA levels in female; **D**: linear regression lines of UA and baPWV stratified by Hcy levels in female

Figure **1** demonstrates the associations of one-SD increase in Hcy among participants grouped by UA status, and the associations of one-SD increase in UA among participants grouped by Hcy status. The effect of Hcy was significant in people with low UA level, and the impact of UA was dominant in those with low Hcy level for males. For females, the effect of Hcy was significant in people with low UA level, while the UA was significantly associated with baPWV regardless of Hcy level. The regression lines after adjusting for confounding factors of Hcy and baPWV stratified by UA status and the regression lines of UA and baPWV stratified by Hcy status are presented in Fig. **2**. The nomogram of arterial stiffness was drawn based on Hcy and UA (Figure S2), and the risk assessment algorithm of Hcy and UA is shown in Table S3.

### Sensitivity and subgroup analyses

**Fig. 3:**
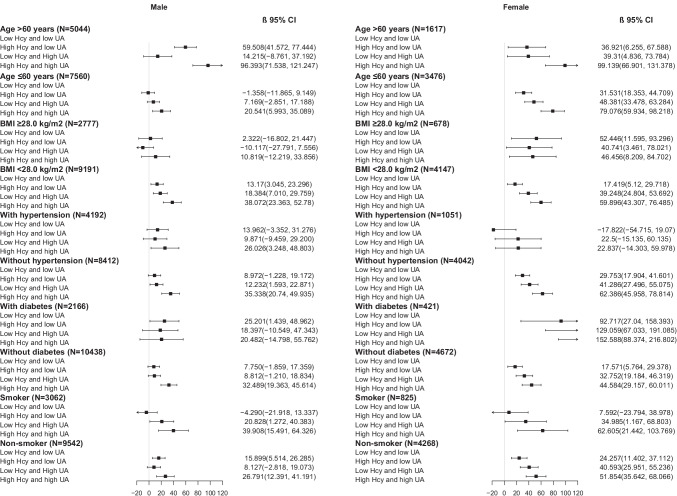
Adjusted mutual associations of serum Hcy and UA with baPWV in subgroups according to age, BMI, hypertension, diabetes, and smoking. Abbreviations: Hcy homocysteine, UA uric acid, baPWV brachial-ankle pulse wave velocity. High level Hcy was defined as > 14 µmol/L in males and > 10 µmol/L in females; and high level UA was defined as > 420 µmol/L in males and > 320 µmol/L in females. Age was divided in to ≤ 60 and > 60 years; BMI was divided in to ≥ 28.0 and < 28.0 kg/m^2^ according to the standard for Chinese population

The consistent results were observed in sensitivity analyses after excluding those with cardiovascular diseases or excluding those using any anti-hypertensive, anti-diabetic, lipid-lowering, anti-gout, or aspirin medications, which could impact the value of Hcy, UA, or baPWV. The detailed results are shown in Table 3. The mutual effect of Hcy and UA in subgroups stratified by age, obesity, hypertension, diabetes, and smoking status is summarized in Fig. **3**. For males, the mutual effects of Hcy and UA were significant in all subgroups, apart from those with obesity or diabetes. For females, the mutual effects were significant in all subgroups, apart from those with hypertension.

**Table 3:** Mutual associations of serum Hcy and UA with baPWV in sensitivity analyses

	Males		Females	
	*β* (95% CI)	*P* value	*β* (95% CI)	*P* value
Sensitivity 1				
(males: 11,977; females: 4949)				
Low Hcy and low UA (males: 6423; females: 2447)				
High Hcy and low UA (males: 2421; females: 1077)	9.559 (0.486, 18.631)	0.039	22.453 (10.541, 34.364)	< 0.001
Low Hcy and High UA (males: 2077; females: 784)	9.675 (0.041, 19.310)	0.049	39.588 (26.105, 53.071)	< 0.001
High Hcy and high UA (males: 1056; females: 641)	31.104 (18.413, 43.795)	< 0.001	53.142 (37.869, 68.414)	< 0.001
Sensitivity 2				
(males: 10,313; females: 4472)				
Low Hcy and low UA (males: 5518; females: 2291)				
High Hcy and low UA (males: 2085; females: 964)	12.451 (2.654, 22.248)	0.013	22.839 (10.692, 34.987)	< 0.001
Low Hcy and High UA (males: 1793; females: 692)	9.965 (− 0.407, 20.338)	0.060	37.633 (23.743, 51.523)	< 0.001
High Hcy and high UA (males: 917; females: 525)	34.165 (20.520, 47.809)	< 0.001	55.306 (39.268, 71.344)	< 0.001

*β* is adjusted for age, education, BMI, physical activity, smoking, drinking, health status, systolic pressure, glucose, triglyceride, HDL-C

Sensitivity 1: Excluding participants with cardiovascular diseases; Sensitivity 2: Excluding participants using medications, including anti-hypertensive, anti-diabetic, lipid-lowering, anti-gout, and aspirin

Abbreviations: *Hcy* homocysteine, *UA* uric acid, *baPWV* brachial-ankle pulse wave velocity

### Mutual effect of Hcy and UA on 10-year cardiovascular risk

In addition, we calculated the 10-year cardiovascular risk for 14,251 individuals between 30 and 74 years old without cardiovascular diseases history. People with both high Hcy and UA had the highest cardiovascular risk, and the OR was 1.49 (95% CI: 1.26–1.76) in males and 7.61 (95% CI: 4.63–12.68) in females (Table 4). Females with high Hcy/low UA or high UA/low Hcy were both associated with increased 10-year cardiovascular risk, and the OR values were 2.85 (95% CI: 1.68–4.84) and 3.10 (95% CI: 1.78–5.38), respectively. Of note, males with high Hcy and low UA were significantly associated with increased cardiovascular risk (OR: 1.30, 95% CI: 1.15–1.47), but not the high UA and low Hcy group (OR: 1.02, 95% CI: 0.90–1.16). The nomogram of 10-year CVD risk was drawn based on Hcy and UA (Figure S3), and the risk assessment algorithm of Hcy and UA is shown in Table S3.

**Table 4:** Mutual association of serum homocysteine and uric acid with 10-year cardiovascular risk ^a^

	Males		Females	
	OR (95% CI)	*P* value	OR (95% CI)	*P* value
Low Hcy and low UA	Reference		Reference	
High Hcy and low UA	1.303 (1.150–1.473)	< 0.001	2.849 (1.683–4.844)	< 0.001
Low Hcy and High UA	1.024 (0.902–1.160)	0.712	3.095 (1.775–5.377)	< 0.001
High Hcy and high UA	1.492 (1.261–1.761)	< 0.001	7.607 (4.629–12.681)	< 0.001

The 10-year risk for men can be calculated as 1 − 0.88936^exp(Σ*βX*−23.9802)^, where *β* is the regression coefficient and *X* is the level for each risk factor as following equation: Σ*βX* = 3.06117*log(age) + 1.12370*log(total cholesterol [mg/dL]) − 0.93263*log(HDL cholesterol [mg/dL]) + 0.65451*smoker [yes or no] + 0.57367*diabetes [yes or no] + 1.93303*log(SBP not treated) or 1.99881*log(SBP treated)

The 10-year risk for women can be calculated as 1 − 0.95012^exp(Σ*βX*−26.1931)^: Σ*βX* = 2.32888*log(age) + 1.20904*log(total cholesterol [mg/dL]) − 0.70833*log(HDL cholesterol [mg/dL]) + 0.52873*smoker [yes or no] + 0.69154*diabetes [yes or no] + 2.76157*log(SBP not treated) or 2.82263*log(SBP treated)

Individuals with 10-year cardiovascular risk ≥ 20% are grouped as high risk

Abbreviations: *Hcy* homocysteine, *UA* uric acid, *SBP* systolic blood pressure

^a^The general cardiovascular risk is calculated using the Framingham Heart Study 10-year risk score. The score includes age, sex, total cholesterol, HDL cholesterol, systolic blood pressure, blood pressure lowering medication use, diabetes status, and smoking status in the primary model

## Discussion

### Paradigm shift from reactive medicine to the advanced PPPM/3 M

This study aimed to investigate the mutual effect of serum Hcy and UA on arterial stiffness in the general population. The results showed that high Hcy or high UA was significantly correlated with increased baPWV level. Individuals with both high Hcy and high UA had the highest level of arterial stiffness and 10-year cardiovascular risk than those with high Hcy or high UA alone. In addition, Hcy and UA have shown potential as biomarkers in the assessment of arterial stiffness and CVD risk. Of note, males of high Hcy and low UA had increased cardiovascular risk, but not high UA and low Hcy, which partly implies males suffer more from Hcy than females, and females are more sensitive to UA than males.

From the perspective of PPPM/3 M, identification of cost-effective and reliable biomarkers is important for the prevention of diseases. The monitoring of these biomarkers could provide predictive information on the onset or prognosis of adverse events. People with high risk should adapt pharmaceutical or non-pharmaceutical interventions to alleviate the progression, which effectively contribute to the paradigm shift from reactive medicine to the advanced approach by utilizing PPPM/3 M concepts [28, 29]. The reactive PPPM/3 M paradigm has been applied in many fields and greatly contributes to the health quality promotion. Golubnitschaja et al. emphasized the basic principle of personalized medicine “one size does not fit all” in BMI management. Individually optimal weight should be clearly discriminated and underlined from the standardized “normal” body weight [30]. Polivka et al. summarized the traditional (sedentary lifestyle, smoking, abnormal alcohol consumption, drug abuse, overweight, hypertension, abnormal sleep patterns) and less explored risk factors (primary vascular dysregulation and associated symptoms characteristic for Flammer syndrome) for young stroke and supported innovative screening programs by application of specialized questionnaires and biomarker panels as well as educational programs adapted to the target population [31]. The risk assessment and patient stratification for breast cancer are emphasized to providing functional links and proposing new approaches from the PPPM/3 M perspective [32]. Innovative screening program is supposed to identify persons in suboptimal health conditions before the clinical onset of metastasizing prostate cancer, and thus to promote the shift of medical paradigm from reactive to advanced PPPM/3M [33]. In previous studies, arterial stiffness is an important risk factor and independent predictor of CVD. BaPWV, as a measure of arterial stiffness, has a great value in guiding clinical treatment and predicting prognosis [34]. In this study, we evaluated the mutual effect of Hcy and UA on arterial stiffness as measured by baPWV to provide a scientific basis for early identification of high-risk population for arterial stiffness, so as to achieve early prediction and primary prevention of arterial stiffness, and proposed the potential mechanism of occurrence and progression of arterial stiffness based on Hcy and UA metabolism.

### Shift from single to mutual effect of Hcy and UA on arterial stiffness

Arterial stiffness is one of the pathological mechanisms of cardiovascular disease and an independent risk factor of all-cause mortality [35]. A prospective nested case–control study showed that high Hcy is significantly associated with an increased risk of stroke [36]. Rotterdam study showed that high UA is an important risk factor of stroke and myocardial infarction [37]. Numerous studies have shown that both Hcy and UA levels are significantly positively correlated with the risk of cardiovascular events [38, 39]. Of note, Hcy and UA are thought to mediate adverse cardiovascular events through arterial stiffness [16, 40]. Previous population-based studies have shown that high Hcy or high UA is an independent risk factor of arterial stiffness [19, 41]. However, studies on the effect of Hcy and UA on arterial stiffness are mostly limited to people with high risk of cardiovascular diseases, such as hypertension, diabetes, and metabolic syndrome. Evidence on the association between serum Hcy, UA, and their combined effects and arterial stiffness in the general population are still lacking. Recently, Wang et al. reported the combined effect of Hcy and UA on subclinical atrial fibrillation [20], and our study supplemented the evidence of the combined effect of Hcy and UA on arterial stiffness.

This study showed that high Hcy and high UA are positively correlated with arterial stiffness in general population after adjusting for confounding factors. Consistent with our findings, Chen et al. found that serum Hcy level is positively correlated with cfPWV level [15]. Wang et al. showed that the baseline level of Hcy is significantly correlated with the increase of cfPWV and crPWV at follow-up [19]. Similarly, several studies have investigated the association between UA and arterial stiffness. Framingham Heart Study showed that serum UA levels are significantly associated with cfPWV and crPWV in the general population [42]. Several prospective cohort studies showed that higher UA level at baseline is significantly associated with arterial stiffness level at follow-up [43]. In this study, we found that individuals with both high Hcy and high UA have the higher level of arterial stiffness than those with high Hcy or high UA alone, suggesting the combined effect of Hcy and UA on arterial stiffness. Wang et al. showed that people with both high Hcy and high UA have the highest risk of SCAF. In addition, the combination of Hcy and UA significantly improves the predictive capacity of SCAF [20]. Furthermore, population-based studies have reported that Hcy level is positively correlated with UA [44], suggesting that vascular damages causing atherosclerotic diseases may be attributed to the combined effect of Hcy and UA.

Vascular endothelium maintains vascular homeostasis by regulating vascular tension, inflammation, and cell growth [45]. Endothelial dysfunction triggers inflammatory activation of endothelial cells, resulting in up-regulation of adhesion molecules and chemokines, and release of cytokines and growth factors to act on adjacent tissues, inducing smooth muscle cell proliferation, vascular wall remodeling and fibrosis, and progression to vascular stiffness [1]. Hcy is a thiol-containing amino acid with cytotoxicity and is an important intermediate product produced in the metabolism of methionine and cysteine [40]. It has been reported that elevated Hcy level can lead to oxidative damage and vascular endothelial dysfunction by reducing methylation [46], reducing endogenous H2S production [47], up-regulating the expression of nicotinamide adenine dinucleotide phosphate (NADPH) oxidase [48], and uncoupling of eNOS [49]. In addition, Hcy can mediate vascular injury through the protein N-homocysteinylation [50]. UA is the end product of human exogenous and endogenous purine metabolism. At the end of purine metabolism, xanthine is oxidized by xanthine oxidase (XO) to form the final product UA [51]. UA is an effective antioxidant component in plasma at physiological concentration [52], but high UA can reduce NO production, induce oxidative stress and inflammatory response by inhibiting eNOS activity, activating the renin-angiotensin system (RAAS) and other pathways [53–55]. Urate crystals produced when serum UA levels exceed the solubility limit induce inflammation and collagen formation, causing vascular damage [56]. On the other hand, UA can cause vascular injury by mediating endothelial dysfunction, and UA is a marker of the up-regulation of XO activity. XO is the main source of reactive oxygen species (ROS) in human body, and the increase of UA level may indicate the oxidative damage caused by XO to vascular endothelium by increasing ROS [57]. Furthermore, Hcy and UA have some shared pathways to affect vascular wall structure and function, such as reducing NO bioavailability by inducing the production of endogenous nitric oxide synthase inhibitor asymmetric dimethylarginine [58, 59], promoting inflammation by activating NF-κB signaling and inducing up-regulation of C-reactive protein (CRP) expression [60, 61], and increasing atherosclerotic disease risk by oxidizing low-density lipoprotein [62, 63]. These results may explain the mutual effect of Hcy and UA on endothelial dysfunction and arterial stiffness. In addition, methionine was converted to adenosine l-homocysteine and further decomposed to homocysteine and adenosine. Part of adenosine was further metabolized to hypoxanthine and entered the UA metabolic pathway, suggesting a potential association between Hcy and UA [16, 20]. Further mechanic studies are needed to clarify the combined role of Hcy and UA in arterial stiffness.

### Stratified analysis of the mutual effects of Hcy and UA on arterial stiffness based on multiple trait subgroups

Stratified analysis showed that Hcy is positively associated with arterial stiffness in people with low UA levels, and UA is positively correlated with arterial stiffness regardless of Hcy level in females, but positively correlated with arterial stiffness only at low Hcy level in males (Hcy < 14 µmol/L). Similarly, Sugiura et al. found that UA is significantly correlated with cardio-ankle vascular index (CAVI) in both genders, and UA has a greater impact in females than males [64]. Fang et al. found that high UA level is positively correlated with the increase of baPWV in females, but not in males [65]. In addition, some studies have reported a stronger association between UA and arterial stiffness in females [16]. The gender difference in the association between UA and arterial stiffness may be caused by the different distribution of Hcy, which also suggests the interactive effect between UA and Hcy. The effect of UA on the risk of arterial stiffness is independent of Hcy in females, whereas the risk of arterial stiffness caused by UA is not significant in males with high Hcy. In addition, Chou et al. found that UA is more strongly associated with insulin resistance and glucose levels in females than males [66]. Hyperglycemia stimulates vascular wall proteins to form advanced glycation end products (AGEs) through non-enzymatic protein glycation, forming irreversible cross-links between proteins [67]. Insulin resistance mediates vascular endothelial dysfunction through decreased NO bioavailability and oxidative stress [68], which partially explains the strong risk of UA-related arterial stiffness.

Golubnitschaja et al. demonstrated that overweight or underweight can increase metabolic load and reduce adaptive capacity through up-regulation of inflammatory signals and increased ROS levels [30]. According to our result stratified by BMI, both high Hcy and UA were significantly positively associated with arterial stiffness in underweight men and overweight and underweight women, suggesting their negative effects on metabolism in the context of abnormal weight (Fig. 3). Flammer Syndrome is a clinical symptom characterized by small body size and low BMI, which is caused by abnormal blood supply due to primary vascular dysregulation [69]. Studies have shown that Flammer Syndrome is closely associated with the progression of various diseases such as glaucomatous optic neuropathy and breast cancer [70]. Whether Flammer Syndrome is the cause of reduced BMI in some people at increased risk of arterial stiffness and whether it is associated with arterial stiffness and CVD need further exploration.

### Association and predictive efficacy of Hcy and UA with 10-year CVD risk

In our subsequent analysis of the association of UA and Hcy with 10-year CVD risk, people with both high UA and Hcy showed the highest risk, supporting our hypothesis that UA and Hcy can mediate CVD by causing arterial stiffness. The study by Zheng et al. suggests that ischemic stroke can be predicted based on routine hematological and biochemical features, and early identification of disease risk may facilitate the formulation of primary care strategies and improve the prognosis of the disease [71]. In this study, the nomogram of CVD risk assessment based on Hcy and UA indicators showed that high Hcy and UA levels predicted the increase of 10-year CVD risk, and the CVD risk was highest when both of them were at high levels. This quantitative risk prediction can identify people at high CVD risk early, and our study suggests that high Hcy and high UA lead to the occurrence of CVD by inducing arterial stiffness. Therefore, timely intervention in the high CVD risk population identified by the model through diet, physical activity, or medication treatment to control the arterial stiffness process early can effectively achieve the primary prevention of CVD. Of note, there was no significantly increased CVD risk in males with high UA and low Hcy, indicating that the impact of Hcy on CVD risk is more obvious than UA in males. Barbieri et al.’s study also showed that high UA level was only associated with severe coronary artery disease in females [72], suggesting the sex difference in UA induced CVD risk. In addition, the findings underline the adverse effect of Hcy on arteriosclerosis and cardiovascular risk and emphasize the clinical importance of combined evaluation and control of Hcy and UA. Hcy and UA provide new targets and insights for the primary prevention and intervention of CVD. People with both high UA and Hcy should pay additional attention to the arterial stiffness susceptibility and CVD risk apart from the instinct metabolic disorders. The dietary behavior and physical activity should be underlined for these people. In addition, this study suggested that Hcy/UA metabolic disorders could cause CVD progression via arterial stiffness, and the biological pathway should be further validated in mechanical researches.

### Comprehensive prevention and control of arterial stiffness

Hcy and UA levels are regulated by both heredity and environmental factors [40, 51]. Improving lifestyle, persisting in physical exercise, ensuring the intake of folic acid and vitamin B12, and reducing the intake of food with high purine content can effectively control the levels of internal Hcy and UA, and thus reduce the arterial stiffness level and cardiovascular risk [73, 74]. Endothelial-dependent vasodilation is effectively improved after treatment with XO inhibitors allopurinol or oxypurinol in patients with coronary artery disease or congestive heart failure [75, 76]. This study indicates the mutual effect of Hcy and UA on arterial stiffness, further suggesting the important role of combined evaluation and control of Hcy and UA in preventing arterial stiffness and cardiovascular events.

## Strength and limitations

This study found the mutual effect of Hcy and UA on arterial stiffness and cardiovascular risk based on a large sample population, and realized early identification of CVD high-risk groups based on Hcy and UA indicators, which contributes to the early comprehensive prevention and control of CVD. The results should be interpreted in the context of some limitations. First, this study is based on a cross-sectional design, and the causal associations of serum Hcy and UA with arterial stiffness cannot be claimed. And the combined effect of Hcy and UA on the dynamic changes of arterial stiffness needs further investigation using longitudinal cohort data. Moreover, the effect of combined control of Hcy and UA on arteriosclerosis and cardiovascular health warrants explanation in clinical trials. Second, the baPWV index measures stiffness of the elastic aorta and the muscular arteries, while the aortic stiffness and muscular stiffness tend to change in the opposite direction with aging. However, baPWV level still enhances the efficacy of predicting cardiovascular events over the Framingham risk score and reflects the cardiovascular risk especially in the Asian population. The association of Hcy and UA with arterial stiffness and screening methods for high-risk CVD patients need further validation in other populations and using other arterial stiffness index, such as cfPWV and the direct measurement of arterial stiffness like elastography. In addition, based on previous studies, we know that the primary prevention and treatment of arterial stiffness and CVD need to be integrated with diet, physical activity, and medication treatment, but how to target the abnormal metabolism of Hcy and UA to comprehensively prevent and treat arterial stiffness needs to be further explored.

## Conclusion and expert recommendations

This study indicates the significant mutual effect of elevated Hcy and UA on arterial stiffness and 10-year cardiovascular risk in the general population. The adverse effect of Hcy on cardiovascular health should be underlined beyond UA, especially for males. Our study suggested the clinical importance of combined evaluation and control of Hcy and UA for preventing arterial stiffness and cardiovascular diseases, and quantified and visualized the risk of arterial stiffness and 10-year CVD by nomogram, which provides a basis for early identification of the progression of arterial stiffness and prediction of CVD risk in general population (Predictive).

Hcy and UA could be novel indicators of arterial stiffness, providing new insights for the early predictive medical approach of CVD risk in the setting of PPPM/3PM health care system. The monitoring of Hcy and UA levels could provide a window opportunity for the primary targeted prevention for the progression of arterial stiffness and adverse CVD events. The health care providers should be aware of the CVD risk for people with Hcy/UA metabolic disorders (Preventive). Besides, pharmaceutic and non-pharmaceutic interventions are needed for alleviating the arterial stiffness progression from the perspective of Hcy and UA using PPPM/3PM strategy, such as balanced diet and physical activity. According to the risk assessment results based on clinical indicators including Hcy and UA, primary prevention or early treatment of arterial stiffness in high-risk groups is of great significance to control the progression of arterial stiffness and reduce the risk of CVD (Personalized medicine).

Overall, there is a mutual effect of Hcy and UA on arterial stiffness and cardiovascular risk, and further studies should investigate the practical benefits of monitoring Hcy and UA levels for cardiovascular health promoting, including the predictive diagnostics, effective prevention, and personalized intervention. This supports the PPPM/3PM strategy and paradigm shift from reactive to advanced in cardiovascular health field. People with both high Hcy and UA should be aware of the cardiovascular health issues at an early stage and get personalized advice on social activity and support, family care, dietary behavior, physical activity, and even the pharmaceutical intervention. Hcy/UA metabolic disorders and cardiovascular adverse events could form a vicious circle, which should be underlined in the advanced PPPM/3PM paradigm.

## Supplementary Information

Below is the link to the electronic supplementary material.

Supplementary file1 (DOCX 226 KB)

## Data Availability

The data and R code used in this current study are available from the corresponding authors on a reasonable request.

## Abbreviations

Hcy: Homocysteine
UA: Uric acid
BHMC: Beijing Health Management Cohort
baPWV: Brachial-ankle pulse wave velocity
eNOS: Endothelial nitric oxide synthase
crPWV: Carotid-radial pulse wave velocity
cfPWV: Carotid-femoral artery pulse wave velocity
SCAF: Subclinical atrial fibrillation
NADPH: Nicotinamide adenine dinucleotide phosphate
XO: Xanthine oxidase
RAAS: Renin-angiotensin system
ROS: Reactive oxygen species
CRP: C-reactive protein
CAVI: Cardio-ankle vascular index
AGEs: Advanced glycation end products
