# Supplementary material for: Mutual effect of homocysteine and uric acid on arterial stiffness and cardiovascular risk in the context of predictive, preventive, and personalized medicine
Source: EPMA J. 2022 Sep 26;13(4):581–95. doi: 10.1007/s13167-022-00298-x (PMC9727018; doi:10.1007/s13167-022-00298-x)
Supplement: Supplementary file 1 — Supplementary file1 (DOCX 226 KB) [file 13167_2022_298_MOESM1_ESM.docx]

**Supplementary file**

**Table S1**: Characteristic in male by homocysteine and uric acid status.

|  | Low Hcy and low UA | High Hcy and low UA | Low Hcy and High UA | High Hcy and high UA |
| --- | --- | --- | --- | --- |
| Participants, n | 6731 | 2563 | 2165 | 1145 |
| Age (years) | 58.06(12.83) | 62.67(15.81) | 55.59(12.44) | 62.22(16.35) |
| Education (n, %) |  |  |  |  |
| Primary school or below | 526(7.8) | 231(9.0) | 168(7.8) | 105(9.2) |
| Middle school | 4009(59.6) | 1566(61.1) | 1286(59.4) | 684(59.7) |
| High school or above | 2196(32.6) | 766(29.9) | 711(32.8) | 356(31.1) |
| Physical activity (n, %) a | 2969(44.1) | 1059(41.3) | 909(42.0) | 438(38.3) |
| Current smoking (n, %) | 1604(23.8) | 663(25.9) | 507(23.4) | 288(25.2) |
| Current drinking (n, %) | 3364(50.0) | 1275(49.7) | 1097(50.7) | 565(49.3) |
| BMI (kg/m2) | 25.72(2.93) | 25.79(3.08) | 26.95(3.14) | 26.67(3.24) |
| Overweight (n, %) b | 1255(19.6) | 519(21.5) | 682(32.8) | 321(29.7) |
| SBP (mmHg) | 127.11(15.62) | 130.33(16.79) | 127.77(14.53) | 131.16(16.21) |
| DBP (mmHg) | 72.74(10.50) | 71.99(10.79) | 74.80(10.62) | 73.55(12.01) |
| Triglyceride (mmol/L) | 1.28[0.94,1.81] | 1.26[0.90,1.81] | 1.71[1.22,2.48] | 1.59[1.14,2.25] |
| Total cholesterol (mmol/L) | 4.51[3.89,5.15] | 4.39[3.80,5.07] | 4.68[4.02,5.30] | 4.56[3.92,5.22] |
| LDL-C (mmol/L) | 2.97[2.40,3.56] | 2.87[2.28,3.51] | 3.08[2.50,3.66] | 3.02[2.38,3.58] |
| HDL-C (mmol/L) | 1.23[1.05,1.44] | 1.20[1.03,1.45] | 1.12[0.98,1.31] | 1.12[0.96,1.32] |
| Fasting glucose (mmol/L) | 5.36[4.97,6.07] | 5.34[4.95,5.97] | 5.40[5.03,5.95] | 5.36[5.00,5.98] |
| HbA1c (%) | 5.77[5.49,6.20] | 5.77[5.48,6.23] | 5.73[5.47,6.08] | 5.82[5.50,6.23] |
| Hypertension | 2054(30.5) | 970(37.8) | 698(32.2) | 470(41.0) |
| Diabetes | 1228(18.2) | 470(18.3) | 288(13.3) | 180(15.7) |
| Dyslipidaemia | 2207(32.8) | 884(34.5) | 1114(51.5) | 556(48.6) |
| Cardiovascular diseases | 308(4.6) | 142(5.5) | 88(4.1) | 89(7.8) |
| Medication use (n, %) |  |  |  |  |
| Antihypertensive | 860(12.8) | 373(14.6) | 308(14.2) | 193(16.9) |
| Antidiabetic | 425(6.3) | 135(5.3) | 88(4.1) | 71(6.2) |
| Lipid lowering | 631(9.4) | 238(9.3) | 155(7.2) | 104(9.1) |
| Anti-gout | 18(0.3) | 17(0.7) | 11(0.5) | 14(1.2) |
| Aspirin | 224(3.3) | 104(4.1) | 49(2.3) | 62(5.4) |
| Homocysteine (μmol/L) | 10.5[9.0,12.0] | 17.1[15.2,21.7] | 10.9[9.5,12.4] | 17.5[15.2,22.2] |
| Uric acid (μmol/L) | 345[307,381] | 352[312,385] | 459[437,494] | 466[438,506] |
| BaPWV (cm/s) | 1471[1337,1674] | 1552[1362,1807] | 1460[1335,1641] | 1575[1393,1822] |

Data are presented as mean (SD), median [IQR] or number (%), as appropriate.

Abbreviations: SD, standard deviation; IQR, interquartile range; BMI, body mass index; SBP, systolic blood pressure; DBP, diastolic blood pressure; HDL-C, high-density lipoprotein cholesterol; LDL-C, low-density lipoprotein cholesterol; HbA1c, glycated haemoglobin; baPWV, brachial-ankle pulse wave velocity.

a Physical activity refers to having moderate or intense exercise ≥80 minutes a weak;

b Obesity is confirmed by BMI ≥28 kg/m^2^.

High level Hcy and UA were defined as >14 µmol/L and >420 µmol/L for male.

**Table S2**: Characteristic in female by homocysteine and uric acid status.

|  | Low Hcy and low UA | High Hcy and low UA | Low Hcy and High UA | High Hcy and high UA |
| --- | --- | --- | --- | --- |
| Participants, n | 2486 | 1102 | 814 | 691 |
| Age (years) | 50.74(11.88) | 56.98(14.60) | 55.90(13.18) | 64.69(15.26) |
| Education (n, %) |  |  |  |  |
| Primary school or below | 214(8.6) | 105(9.5) | 59(7.2) | 74(10.7) |
| Middle school | 1437(57.8) | 630(57.2) | 525(64.5) | 416(60.2) |
| High school or above | 835(33.6) | 367(33.3) | 230(28.3) | 201(29.1) |
| Physical activity (n, %) ^a^ | 885(35.6) | 378(34.3) | 317(38.9) | 262(37.9) |
| Current smoking (n, %) | 386(15.5) | 179(16.2) | 151(18.6) | 109(15.8) |
| Current drinking (n, %) | 922(37.1) | 388(35.2) | 298(36.6) | 250(36.2) |
| BMI (kg/m^2^) | 23.58(3.14) | 23.97(3.32) | 25.57(3.75) | 26.09(3.67) |
| Overweight (n, %) ^b^ | 216(9.2) | 120(11.5) | 164(20.9) | 178(27.4) |
| SBP (mmHg) | 115.97(16.68) | 120.44(18.08) | 123.83(16.72) | 128.13(18.97) |
| DBP (mmHg) | 67.05(9.86) | 67.56(9.59) | 70.77(10.33) | 68.75(10.64) |
| Triglyceride (mmol/L) | 1.02[0.77,1.40] | 1.07[0.81,1.45] | 1.42[0.99,1.99] | 1.41[1.04,2.00] |
| Total cholesterol (mmol/L) | 4.73[4.16,5.34] | 4.76[4.22,5.37] | 4.82[4.24,5.51] | 4.78[4.21,5.53] |
| LDL-C (mmol/L) | 3.02[2.48,3.58] | 3.05[2.51,3.65] | 3.16[2.56,3.75] | 3.10[2.51,3.80] |
| HDL-C (mmol/L) | 1.48[1.24,1.74] | 1.51[1.26,1.79] | 1.33[1.12,1.57] | 1.32[1.11,1.62] |
| Fasting glucose (mmol/L) | 5.00[4.71,5.36] | 5.00[4.72,5.37] | 5.20[4.83,5.75] | 5.26[4.92,5.76] |
| HbA1c (%) | 5.62[5.38,5.87] | 5.70[5.45,5.97] | 5.84[5.56,6.11] | 5.89[5.65,6.22] |
| Hypertension | 325(13.1) | 236(21.4) | 215(26.4) | 275(39.8) |
| Diabetes | 126(5.1) | 79(7.2) | 102(12.5) | 114(16.5) |
| Dyslipidaemia | 482(19.4) | 223(20.2) | 289(35.5) | 247(35.7) |
| Cardiovascular diseases | 39(1.6) | 25(2.3) | 30(3.7) | 50(7.2) |
| Medication use (n, %) |  |  |  |  |
| Antihypertensive | 129(5.2) | 86(7.8) | 84(10.3) | 142(20.5) |
| Antidiabetic | 32(1.3) | 33(3.0) | 31(3.8) | 43(6.2) |
| Lipid lowering | 119(4.8) | 91(8.3) | 78(9.6) | 84(12.2) |
| Anti-gout | 1(0.0) | 1(0.1) | 0(0.0) | 1(0.1) |
| Aspirin | 21(0.8) | 27(2.5) | 23(2.8) | 24(3.5) |
| Homocysteine (μmol/L) | 7.8[6.6,8.9] | 11.8[10.8,13.8] | 8.3[7.3,9.2] | 12.1[11.0,14.3] |
| Uric acid (μmol/L) | 258[226,287] | 266[234,292] | 350[333,381] | 364[339,410] |
| BaPWV (cm/s) | 1322[1206,1468] | 1423[1267,1632] | 1465[1318,1651] | 1593[1411,1818] |

Data are presented as mean (SD), median [IQR] or number (%), as appropriate.

Abbreviations: SD, standard deviation; IQR, interquartile range; BMI, body mass index; SBP, systolic blood pressure; DBP, diastolic blood pressure; HDL-C, high-density lipoprotein cholesterol; LDL-C, low-density lipoprotein cholesterol; HbA1c, glycated haemoglobin; baPWV, brachial-ankle pulse wave velocity.

a Physical activity refers to having moderate or intense exercise ≥80 minutes a weak;

b Obesity is confirmed by BMI ≥28 kg/m^2^.

High level Hcy and UA were defined as >10 µmol/L and >320 µmol/L for female.

**Table S3**: Risk assessment algorithm of serum uric acid and homocysteine.

| Arterial stiffness risk stratification | ${\frac{e^{0.32195*highUA+0.47653*highHOMO+0.18274}}{1+e^{0.32195*highUA+0.47653*highHOMO+0.18274}}}$ |
| --- | --- |
| Cardiovascular 10-year risk assessment | $\frac{e^{0.12146*highUA+0.22002*highHOMO-1.65842}}{1+e^{0.12146*highUA+0.22002*highHOMO-1.65842}}$ |

HighUA was defined as serum uric acid >420 µmol/L for male and >320 µmol/L for female; HighHOMO was defined as serum homocysteine >14 µmol/L for male and >10 µmol/L for female.


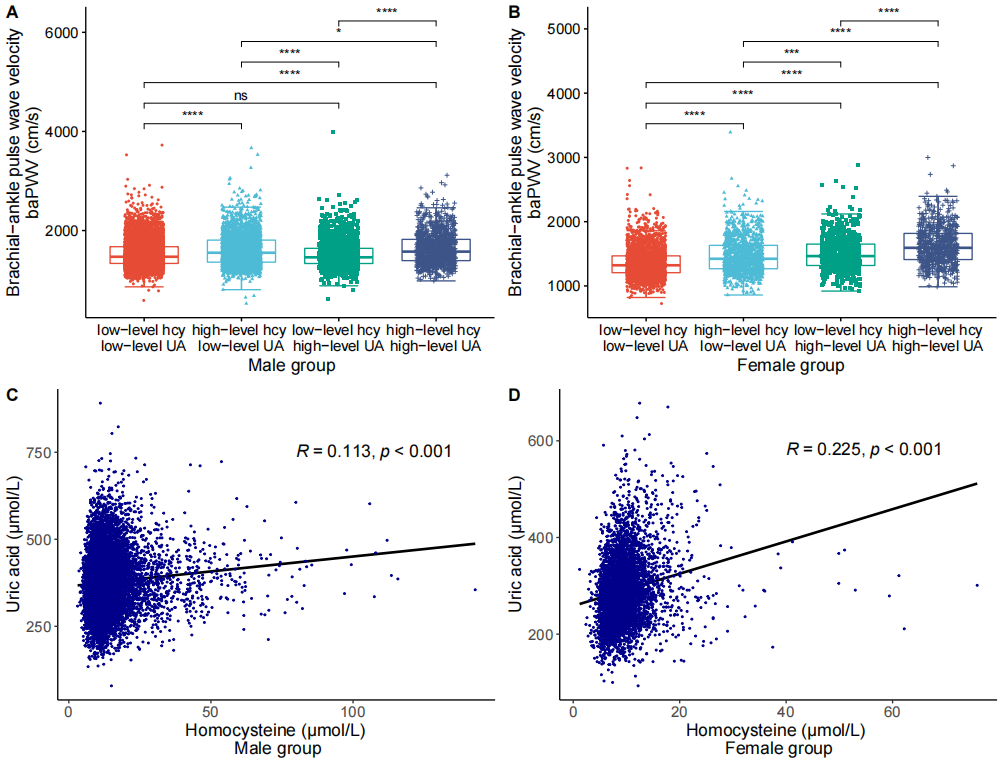


**Figure S1:** Distribution plot of baPWV and the correlation of serum Hcy and UA.

Abbreviations: Hcy, homocysteine; UA, uric acid; baPWV, brachial-ankle pulse wave velocity.

1. B: Box-plot of baPWV according to Hcy and UA status.

C-D: Correlation plot of serum Hcy and UA in male and female.


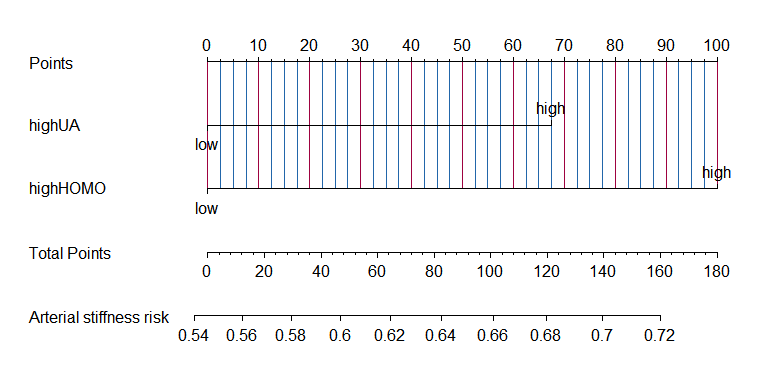


**Figure S2:** Arterial stiffness risk stratification by combined assessment of serum uric acid and homocysteine.

HighUA was defined as serum uric acid >420 µmol/L for male and >320 µmol/L for female; HighHOMO was defined as serum homocysteine >14 µmol/L for male and >10 µmol/L for female.


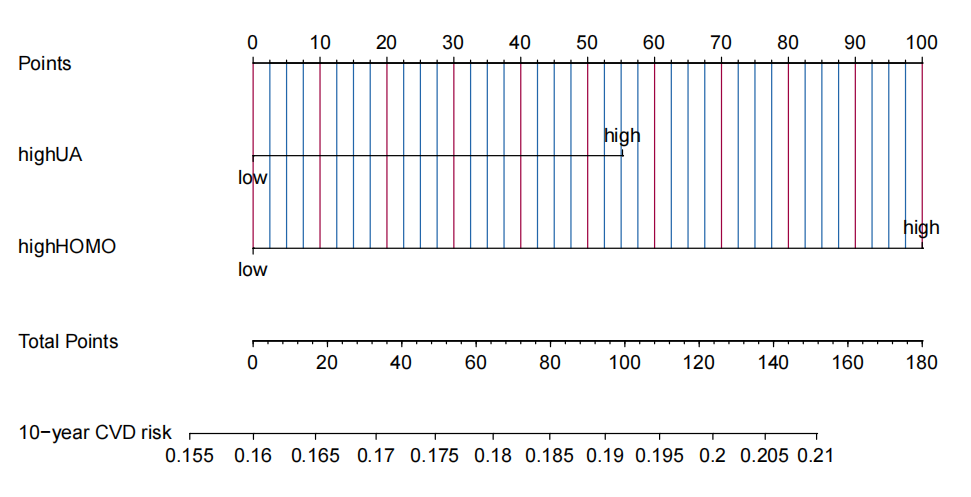


**Figure S3:** Cardiovascular 10-year risk stratification by combined assessment of serum uric acid and homocysteine.

HighUA was defined as serum uric acid >420 µmol/L for male and >320 µmol/L for female; HighHOMO was defined as serum homocysteine >14 µmol/L for male and >10 µmol/L for female.
